# Reconstruction and Analysis of a Genome-Scale Metabolic Model of *Acinetobacter lwoffii*

**DOI:** 10.3390/ijms25179321

**Published:** 2024-08-28

**Authors:** Nan Xu, Jiaojiao Zuo, Chenghao Li, Cong Gao, Minliang Guo

**Affiliations:** 1 College of Bioscience and Biotechnology, Yangzhou University, Yangzhou 225009, China; nanxu@yzu.edu.cn (N.X.);; 2 School of Biotechnology, Jiangnan University, Wuxi 214122, China

**Keywords:** *Acinetobacter lwoffii*, genome-scale metabolic model, phenol degradation, drug targets

## Abstract

*Acinetobacter lwoffii* is widely considered to be a harmful bacterium that is resistant to medicines and disinfectants. *A. lwoffii* NL1 degrades phenols efficiently and shows promise as an aromatic compound degrader in antibiotic-contaminated environments. To gain a comprehensive understanding of *A. lwoffii*, the first genome-scale metabolic model of *A. lwoffii* was constructed using semi-automated and manual methods. The *i*NX811 model, which includes 811 genes, 1071 metabolites, and 1155 reactions, was validated using 39 unique carbon and nitrogen sources. Genes and metabolites critical for cell growth were analyzed, and 12 essential metabolites (mainly in the biosynthesis and metabolism of glycan, lysine, and cofactors) were identified as antibacterial drug targets. Moreover, to explore the metabolic response to phenols, metabolic flux was simulated by integrating transcriptomics, and the significantly changed metabolism mainly included central carbon metabolism, along with some transport reactions. In addition, the addition of substances that effectively improved phenol degradation was predicted and validated using the model. Overall, the reconstruction and analysis of model *i*NX811 helped to study the antimicrobial systems and biodegradation behavior of *A. lwoffii.*

## 1. Introduction

*Acinetobacter lwoffii* is a Gram-negative bacterium of the genus Acinetobacter, class Gammaproteobacteria. While *Acinetobacter baumannii* is the most common cause of infections among 38 species of this *Acinetobacter* genus [1], there have been increasing reports that *A. lwoffii* has been identified as a serious human pathogen. *A. lwoffii* causes nosocomial infections, such as bacteremia, pneumonia, and meningitis [2]. *A. lwoffii* possesses resistance to multiple drugs and disinfectants, which allows it to survive in clinical and hospital environments. Whole-genome sequencing on clinical isolates of *A. lwoffii* has been used to annotate antibiotic resistance genes [3]. Comparative genomics studies of the phylogeny of *A. lwoffii* and other *Acinetobacter* strains [4,5,6] have detected more resistance genes on plasmids in *A*. *lwoffii* strains compared to *A*. *baumannii* strains [7]. The challenge of eliminating bacterial cells in the environment is exacerbated by multidrug resistance, which renders infections more harmful. Although it has not yet been fully utilized, metabolism is a promising source of possible medication adjuvants for harmful bacteria [8]. A systems-level framework would help to explore the relationship between microbial metabolism and antimicrobials, and to identify drug targets for treating the pathogens [9].

In addition to pathogenicity and drug resistance, the biodegradation potential of *A. lwoffii* is currently recognized. Like other *Acinetobacter* bacteria, *A. lwoffii* is ubiquitous in soil and water environments, as well as in animal and human clinical samples. For example, *A. lwoffii* DNS32 can degrade atrazine (100 mg/L) [10], and its degradation capacity has been optimized in a microbial consortium [11]. Adaptive laboratory evolution produced *A. lwoffii* NL115, capable of degrading 1500 mg/L phenol [12]. *A. lwoffii* MG04 can degrade pyrethroids [13]. Environmental strains of *A. lwoffii* contain heavy metal resistance genes, allowing the organism to survive in high concentrations of heavy metals [14]. A system-level platform would help to uncover the metabolic mechanisms underlying biodegradation, and to design optimal conditions that would enhance biodegradation processes.

Genome-scale metabolic models (GEMs) are effective systemic platforms for elucidating the associations among metabolites, genes, and reactions in living organisms [15]. For the genus *Acinetobacter*, five models described for *A. baumannii* and one for *A. baylyi* have been used to explore microbial growth phenotypes, essential metabolism, antimicrobial development, metabolic alterations under certain conditions, and other metabolic characteristics [16,17,18,19,20,21]. Despite the large number of genomes and draft sequences of *A. lwoffii*, a systematic and comprehensive metabolic model is still lacking. In this study, we describe the reconstruction of the first genome-scale metabolic model, designated *i*NX811. *A*. *lwoffii* NL1 is resistant to multiple compounds and effectively degrades phenol [22]. The application of the model *i*NX811 was in the two relevant metabolic and physiological features. The essential metabolism of *A. lwoffii* was analyzed to identify in silico drug targets. The metabolic response under phenol stress was evaluated by the integration of transcriptomic data into the model *i*NX811. The addition of TCA cycle intermediates and alanine to improve phenol degradation was predicted and validated using constraint-based analyses and biochemical studies.

## 2. Results and Discussion

### 2.1. Characteristics of the Genome-Scale Metabolic Model iNX811

**Figure 1:**
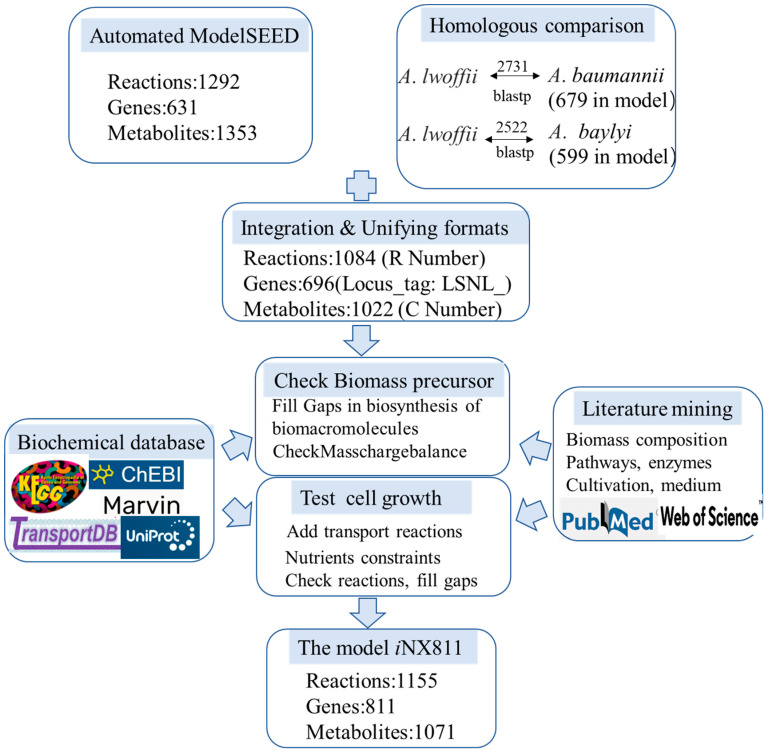
The genome-scale model reconstruction process for *A. lwoffii*.

**Figure 2:**
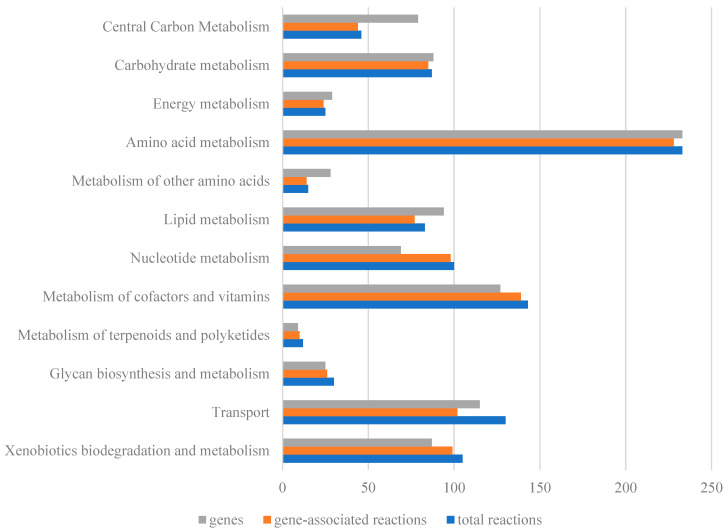
Genes, reactions, and gene-associated reactions for each metabolic subsystem of the model *i*NX811. Genes indicate how many genes there are in each metabolic subsystem, total reactions show how many reactions there are in each metabolic subsystem, and gene-associated reactions show which metabolic reactions have their encoding genes annotated.

As shown in Figure 1, reconstruction of the genome-scale metabolic model for *A. lwoffii* mainly included three steps: (1) The ModelSEED model for *A. lwoffii* NL1 was built using annotation findings from the RAST server [23], and it included 1292 reactions, 1353 metabolites, and 631 genes. Moreover, 679 and 599 genes of *A. lwoffii* NL1 were matched to the protein sequences in the models *i*ATCC19606 and *i*AbaylyiV4, respectively, and utilized to extract model contents from the two models based on gene–protein–reaction relationships. (2) After combining the SEED model and homologous models, metabolites and gene names were unified into their own formats, and duplicate reactions were removed. The draft model was modified trivially and manually, and the improved draft model had 696 genes, 1022 metabolites, and 1084 reactions. (3) Metabolic gaps in the biosynthesis pathways for biomass components were filled using the literature, biochemical databases, and gene reannotation. After passing biomassPrecursorCheck in the COBRA toolbox, 102 transporters from *A. lwoffii* NL1 were introduced to the model, with nutrient uptake rates serving as constraints. Then, growth phenotypes were modeled and compared to the experimental data. Iterative manual curation of the disagreements was required to fill in metabolic gaps, change the direction of reactions, and correct incorrectly anticipated reactions. Finally, there were 811 genes, 1071 metabolites, and 1155 reactions in the model *i*NX811 (Tables S1 and S2 and Supplementary File S2), which had a 52% total MEMOTE score [24] (Supplementary File S3). This model accounts for 76 metabolic pathways in 16 metabolic subsystems, including the biosynthesis and biodegradation of primary and secondary metabolites. Except for exchange, tRNA biosynthesis, general reactions, and biomass formation, the other 12 metabolic subsystems are generally shown in Figure 2. The average ratio of gene–protein–reaction (GPR) association reactions per subsystem was 92.8%, reflecting adequate genome annotation using the model *i*NX811. Amino acid metabolism comprised the highest number of reactions and genes, consistent with the capability of *A. lwoffii* to synthesize 20 amino acids. Xenobiotic biodegradation and metabolism had 103 reactions, with 94.3% GPR associations. They included 23 chlorocyclohexane and chlorobenzene degradation reactions, 17 xylene degradation reactions, 17 benzoate degradation reactions, 13 fluorobenzoate degradation reactions, 13 toluene degradation reactions, 5 reactions each for aminobenzoate, polycyclic aromatic hydrocarbon, and atrazine degradation, and 5 scatter reactions. The findings revealed genes with potential value in degrading a wide range of xenobiotics.

To identify the characteristics of the model *i*NX811, genome-scale metabolic models of *A. baumannii i*ATCC1960 [15] and *A. baylyi* ADP1 [20] were compared. The coverage of the annotated open reading frames of the three models was approximately 23.2% (*i*NX811), 23.4% (*i*ATCC1960), and 23.1% (ADP1). A total of 468 metabolites shared by the three models comprised 518 metabolic reactions, excluding exchange reactions. These represent the core metabolism of *Acinetobacter*, including common primary metabolism, ten reactions in the metabolism of terpenoids and polyketides, and ten xenobiotic metabolic reactions (Table S3). In the model *i*NX811, 800 and 495 metabolites coexisted in the *i*ATCC19606 and *A. baylyi* ADP1 models (Table S3), respectively, which had similar trends to the genome homology of *A. lwoffii* with *A. baumannii* (74.13%) and *A. baylyi* (68.3%). Compared to the other two models, 64 unique metabolites in the model *i*NX811 participated in 65 unique reactions (Table S4), excluding exchange reactions. They were mainly distributed in xenobiotic biodegradation and metabolism (44 reactions), as well as amino acid and alternative carbon metabolism.

### 2.2. Model Validation by Cell Growth Phenotypes and Genotypes

The utilization of 39 carbon and nitrogen sources was simulated using the model *i*NX811 (Table 1). The in silico growth coincided well with the experimental results, except for when glycerol was employed as the carbon source. *A. lwoffii* NL1 did not utilize glycerol as the sole carbon source, which may have been related to over-reduction. Adaptive laboratory evolution could overcome this barrier and enable in silico growth on glycerol MM medium [25]. This model could reflect the metabolic pathways of various carbon and nitrogen sources, including common sugars, amino acids, alcohols, carboxylic acids, and uncommon aromatic xenobiotics. The carbon source spectrum of *A. lwoffii* is relatively broad, and the metabolites did not support cell growth, mainly because of the loss of relevant transporters or metabolic enzymes. For example, *A. lwoffii* NL1 can grow on fructose, but not on glucose, because the hexokinase in *A. lwoffii* NL1 cannot convert glucose to glucose-6-phosphate. These intermediates in glycolysis and the TCA cycle, such as acetate, ethanol, citrate, and succinate, can support cell growth as sole carbon sources. In addition, the observation that *A. lwoffii* NL1 could use simple phenols and phenolic acids as sole carbon sources suggests that *A. lwoffii* has a good biodegradability capacity [26,27,28].

In silico single-gene deletions were induced using the model *i*NX811. Compared with the essential genes identified by transposon insertion mutants, the prediction accuracy of essential genes was 79.6% on LB medium and 75.9% on succinate as the sole carbon source. The high accuracy of essential gene prediction validated that the model *i*NX811 can reflect the metabolic characteristics of *A. lwoffii* NL1. Precisely 100 genes were predicted to be essential for cell growth in the LB medium (Table S4). Of these genes, 95 were validated as essential in the DEG database and participated in 175 biochemical reactions involving 34 metabolic pathways. A total of 164 genes were predicted to be essential for cell growth with succinate as the sole carbon source (Table S4). Among these, 157 genes (95.7%) were validated in the DEG database and participated in 270 biochemical reactions involving 41 metabolic pathways.

### 2.3. Essential Metabolite Analysis for In Silico Drug Targets

*A. lwoffii* is an opportunistic pathogen that causes nosocomial infections in humans [3]. A recent study found that the NL1 strain has numerous antibiotic resistances [21], making it difficult to kill the bacterial cells. As a result, identifying feasible therapeutics is an urgent and substantial task [29]. One approach to address this issue has been to use systems-level computational models, specifically genome-scale metabolic models [30]. Pathogenic bacteria’s GEMs have recently been reconstructed, revealing genes and pathways that are essential to the survival and spread of zoonotic and human infections [31,32,33]. GEMs, as powerful computational tools, can predict cellular essential genes, reactions, and metabolites. Essential metabolites are required for cellular growth, and their absence leads to cell death. Therapeutic targets can be created based on the structural analogues of essential metabolites of pathogens, hence eliminating the need for labor-intensive random chemical library screening. The essential metabolite filtering method (EMFilter) was used for discovering effective drug targets by removing currency metabolites, metabolites consumed by only one outgoing reaction, metabolites present in human GEMs, and the essential metabolites consumed by homologous enzymes with human genomes. The EMFilter procedure was employed in developing therapeutic strategies against various pathogens, such as *A. baumannii* [18], *Vibrio vulnificus* [34], and *Cryptococcus neoformans* [35]. Using the validated model *i*NX811, eighty-five genes were predicted to be essential for cell growth on arbitrary complex media (see Table S7) and were related to 148 reactions and 216 metabolites. According to the EMFilter procedure [18], 34 currency metabolites, 85 metabolites involved in only one consumption reaction, and 138 metabolites in the human metabolic model Recon3D were eliminated. Twelve of the essential metabolites identified were predicted to be potential drug targets (Table 2). These were mainly distributed in glycan biosynthesis, lysine biosynthesis, and the metabolism of cofactors and vitamins.

Specifically, four essential metabolites (D-glucosamine 1-phosphate, D-arabinose 5-phosphate, dTDP-glucose, and alpha-trehalose 6-phosphate) were involved in glycan biosynthesis, which affects the cell wall and membrane structures of *A. lwoffii*. D-glucosamine 1-phosphate 1,6-phosphomutase (GlmM) catalyzes the reversible isomerization of D-glucosamine 1-phosphate and D-glucosamine 6-phosphate. The phosphoglucosamine mutase (GlmM) in *Legionella pneumophila* was discovered as a candidate therapeutic target enzyme after docking the substrate for the most favorable binding of S-mercaptocystein [36]. Glucosamine-1-phosphate-acetyltransferase (GlmU) is involved in bacterial cell wall biosynthesis, making it an appealing target for creating antibacterial drugs for *Aspergillus terreus* [37] and *Haemophilus influenzae* [38]. D-arabinose-5-phosphate can be reversibly converted to d-ribulose 5-phosphate by D-arabinose-5-phosphate isomerase, which has been utilized as an antibacterial target in *Bacteroides fragilis* [39], *Pseudomonas aeruginosa* [40], and *Burkholderia pseudomallei* [41]. D-arabinose-5-phosphate and phosphoenolpyruvate form 3-deoxy-d-manno-octulosonate 8-phosphate, which is a precursor of lipopolysaccharides that are necessary for the growth and virulence of *Acinetobacter* [42]. dTDP-Glucose synthase (glucose-1-phosphate thymidylyltransferase) was predicted to be a drug target for *A. lwoffii*, having previously been identified as a possible target for antibacterial medicines in *M. tuberculosis* [43] and *P. aeruginosa* [44]. It has been found that, in pathogenic fungi like *Candida albicans*, dTDP-glucose 4,6-hydrolyase preserves cell wall integrity and virulence [45]. dTDP-Glucose 4,6-dehydratase from *Streptococcus* mutants (serotype c) was found in the DrugBank database (www.drugbank.com). Trehalose 6-phosphate has been discovered as an essential metabolite of *A. lwoffii*. Some potential drug targets were found in trehalose metabolism [46]. Trehalose 6-phosphate synthase, for example, has been investigated as an antifungal target in *C. albicans* [47] and *Cryptococcus neoformans* [48].

Four essential metabolites (dehydrodipicolinate, N-succinyl-LL-2,6-diaminoheptanedioate, meso-2,6-diaminoheptanedioate, and L-Aspartate 4-semialdehyde) are involved in the lysine biosynthesis of *A. lwoffii*. The lysine biosynthesis pathway is of special interest in pharmacology because the absence of DAP in mammalian cells allows for using lysine biosynthesis genes as bacteria-specific drug targets [49]. Dehydrodipicolinate synthase (DHDPS), dihydrodipicolinate reductase (DapB), diaminopimelate epimerase (DapF), succinyldiaminopimelate transaminase, succinyl-diaminopimelate desuccinylase (DapE), UDP-N-acetylmuramoyl-L-alanyl-D-glutamate-2,6-diaminopimelate ligase (MurE), aspartate-semialdehyde dehydrogenase (ASADH), and 4-hydroxy-tetrahydrodipicolinate synthase (DapA) are relevant enzymes that catalyze reactions containing these essential metabolites. Potential antibacterials, inhibitors of DHDPS, were created for *Bacillus anthracis* [50] and *Campylobacter jejuni* [51]. Studies on DapB inhibition were conducted on *M. tuberculosis* [52] and *Staphylococcus aureus* [53]. Research has been carried out on the importance of DapF as a therapeutic target in *Bordetella pertussis* [54] and *Enterococcus faecalis* [55]. Succinyldiaminopimelate transaminase in *M. tuberculosis* was studied to develop novel antibacterial medications [56]. DapE has emerged as a promising bacterial enzyme target, and its related inhibitors have been identified in *H. influenzae* [57], *A. baumannii* [58], and a few pathogens [59]. *A. baumannii* employed MurE, which was found in the DrugBank database (www.drugbank.com), as a therapeutic target [60]. The inhibition of ASADHs has been discussed as a possible therapeutic target for producing novel antibacterial drugs [61]. The structures of DapA from *M. tuberculosis* [62], *P. aeruginosa* [63], and *S. aureus* [64] were examined to develop drugs that inhibit the DapA family.

In folate biosynthesis, the essential metabolites 4-aminobenzoate and 2-amino-4-hydroxy-6-hydroxymethyl-7,8-dihydropteridine were also found in the opportunistic pathogen *V. vulnificus*, and structural analog 24837 of 4-aminobenzoate has antibacterial effects [34]. Chorismate, as a popular pharmacological target, has been effectively employed against pathogenic bacteria [65,66]. 1-Deoxy-D-xylulose 5-phosphate, predicted as the essential metabolite, was involved in the metabolism of thiamine and vitamin B6. Related enzymes, 1-deoxy-D-xylulose-5-phosphate synthase and pyridoxine 5′-phosphate synthase, were predicted to be drug targets in pathogens [67,68,69]. Therefore, essential metabolite analysis can aid in predicting possible pharmacological targets for antimicrobial treatments. Some metabolites and enzymes have been shown to be efficient targets in pathogenic bacteria or fungi. The inhibition of pathogens illustrates the GEM’s utility in discovering therapeutic targets, making it exceedingly viable to produce antimicrobials against *A. lwoffii* in future studies.

### 2.4. Integration of Transcriptomic Data in the Genome-Scale Metabolic Model

**Figure 3:**
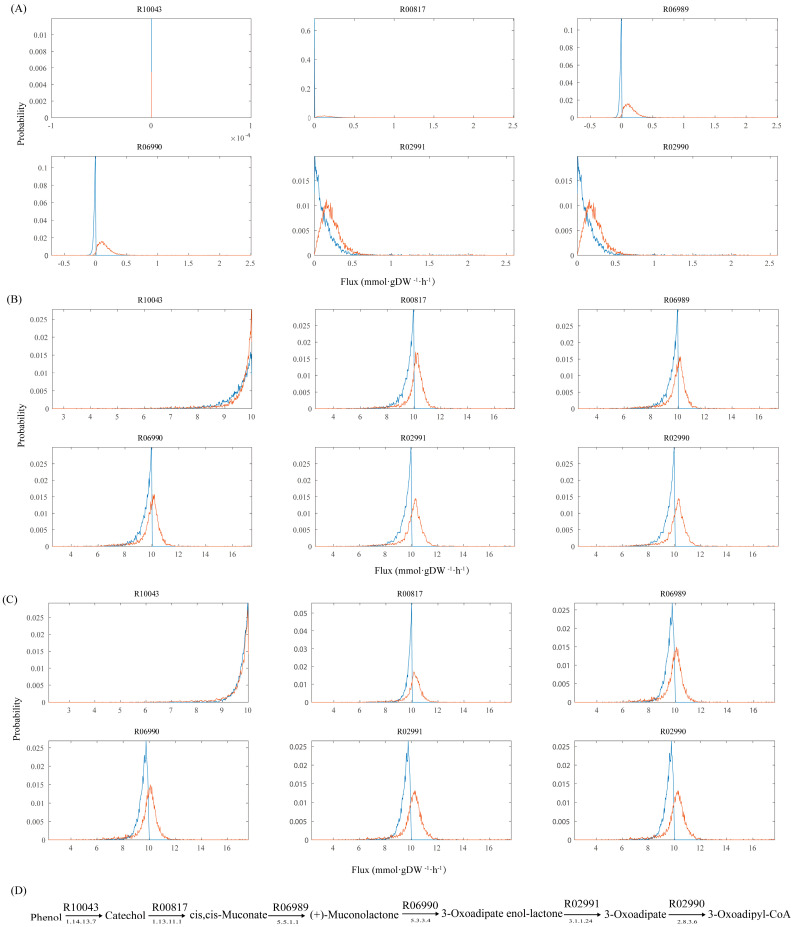
The metabolic states feasible through the phenol *ortho*-cleavage pathway of *A. lwoffii* under different cultivation conditions: (**A**–**C**) Different cultivation conditions, i.e., NaAc as the sole carbon source, NaAc with 0.5 g/L phenol as carbon sources, and NaAc with 1.5 g/L phenol as carbon sources. The range of flux distributions with and without the regulatory constraints is shown in blue and in orange, respectively. (**D**) The phenol *ortho-cleavage* pathway in *A. lwoffii*. All of the reactions (R numbers) were from the KEGG database.

**Figure 4:**
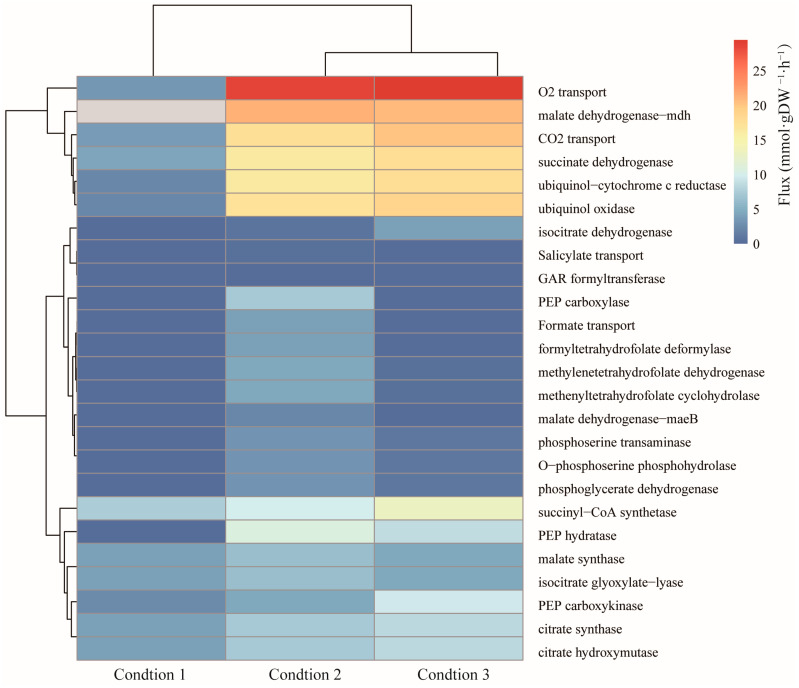
Flux comparison of three context-specific genome-scale models constrained by differential transcriptomic data. Conditions 1–3 involved using NaAc as the only carbon source, adding 0.5 g/L of phenol as a carbon source, and adding 1.5 g/L of phenol as a carbon source, respectively. GAR, phosphoribosylglycinamide; PEP, phosphoenolpyruvate.

*A. lwoffii* NL1 is one of the most efficient degraders of phenols. GEMs for microbial biodegradation were reconstructed to analyze the degradation pathways of non-conventional substrates [70,71], to improve biodegradation by modeling-based identification of media supplements [72,73], to predict degradation behavior by imposing more constraints [74], and to simulate interactions between degraders and non-degraders in consortium systems [75]. Modern omics data integrated into GEMs contribute to the identification of novel metabolic insights into this microbe and are increasingly being utilized to promote a better understanding of complex biological systems [76,77]. In this study, the Gene Inactivity Moderated by Metabolism and Expression (GIMME) algorithm [78] was used to construct context-specific GEMs of *A. lwoffii*. Gene expression data of *A. lwoffii* on sodium acetate (NaAc) as the sole carbon source and NaAc with 0.5 or 1.5 g/L phenol as a carbon source were integrated into the model *i*NX811, and the three relevant context-specific GEMs included 1050 reactions and 1016 metabolites, 1045 reactions and 1017 metabolites, and 1077 reactions and 1016 metabolites, respectively (see Supplementary File S4). After introducing transcriptional constraints, the reaction flux of the phenol *ortho*-cleavage pathway was narrowed under the three environmental conditions (Figure 3) using random sampling. Integrating transcriptomic data into the model *i*NX811 reduces the number of metabolic states [79]. The metabolic flux of the three context-specific GEMs was compared to investigate the metabolic differences under phenol stress. Reactions exhibiting a significant flux change (added phenol/without phenol) were assumed to change under phenol stress (Figure 4). Specifically, the in silico metabolic flux for complexes II and IV of oxidative phosphorylation and D-phosphoglycerate 2,3-phosphomutase in glycolysis and the conversion of acetyl-CoA to malate (citrate synthetase, citrate hydroxymutase, isocitrate dehydrogenase, succinyl-CoA synthetase, succinate dehydrogenase, and fumarase) in the TCA cycle were enhanced, suggesting accelerated energy conversion in response to phenol stress. The conversion between 3-phosphoglycerate and phosphoenolpyruvate, the glyoxylate cycle, and the outflow from malate to pyruvate were downregulated under greater phenol stress. The oxaloacetate anaplerotic reaction became active under high phenol stress with increased phosphoenolpyruvate (PEP) carboxykinase and decreased PEP carboxylase. Although no salicylate was present in the medium, the expression of the salicylate exporter was activated under phenol stress, which might weaken the competition for catechol metabolism in the beta-ketoadipate branch. Under phenol stress, there was an increase in CO_2_ permeability and oxygen absorption level. It was found that *Pseudomonas* CF600 in continuous culture responded significantly to oxygen in terms of phenol consumption [80]. The flux from 3-phosphoglycerate to serine decreased. Folate downregulates one carbon pool as the phenol concentration increases [81]. The transcriptome-constrained GEMs may be used to investigate reactions with large flux changes at different phenol levels, allowing us to gain a better understanding of the metabolic response to phenol stress in *A. lwoffii* and identify potential metabolic modules to enhance biodegradation.

### 2.5. Prediction of Targets for Increased Phenol Degradation

**Figure 5:**
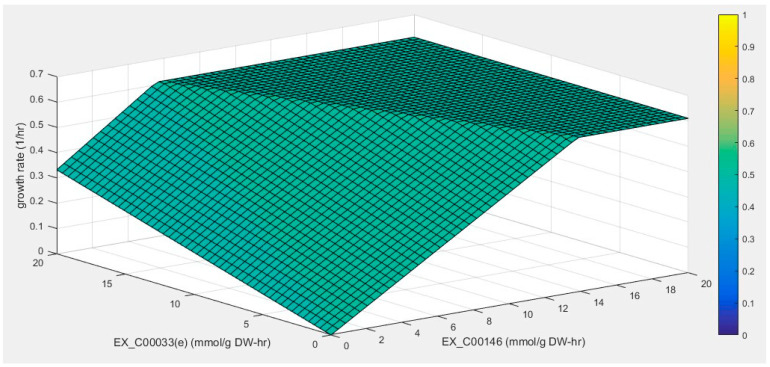
Effects of phenol and sodium acetate on cell growth using phenotype phase-plane analysis. The Ex_C00146 and Ex_C00033(e) axes represent the phenol uptake rate and sodium acetate uptake rate, respectively. The colored legend signifies cell growth rates.

Furthermore, the model *i*NX811 was used to find some exogenous substances capable of improving phenol breakdown by simulating the interaction between phenol and supplement consumption and cell growth. Exogenous substances that benefit phenol degradation were selected from the test carbon sources of *A. lwoffii*. As previously reported, adding sodium acetate can shorten the time taken to reach maximum biomass compared to its growth on phenol as the sole carbon source (Figure S1). The phenotype phase-plane analysis was used to investigate the interaction of the two substrate utilizations and their effects on cell growth. Two distinct surfaces (I and II) are shown in Figure 5. In plane I, cell growth was affected by the uptake of both substrates. Cell growth increased linearly with the increase in sodium acetate or phenol when the uptake rate of the other substrate was zero. When one parameter was fixed, cell growth increased with the uptake rate of other substrates. Cell growth reached its maximum value on the two-phase boundary line. In plane II, cell growth remained unchanged with the uptake of the two substrates. The metabolic traits of available carboxylic acids, saccharides, and amino acids were investigated using the model *i*NX811. The PHPP results for certain carbon sources exhibited the same mode as that of sodium acetate (Figure S2A–O), which theoretically benefits cell growth in the presence of phenol. Fructose, pyruvate, malate, and succinate were chosen as candidates based on the PHPP results.

**Figure 6:**
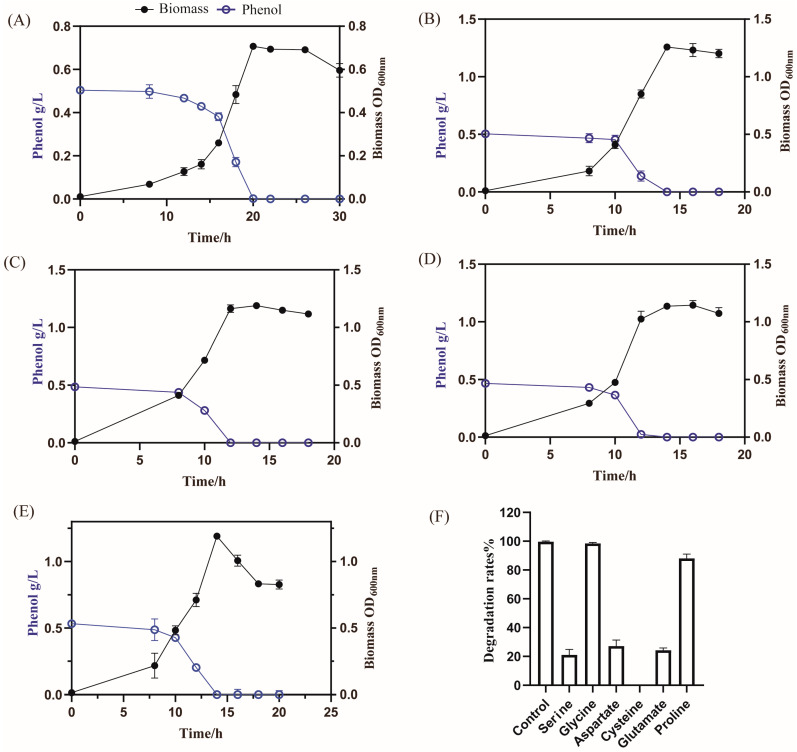
Effects of the addition of various substances on phenol degradation: (**A**–**E**) the dynamic curves of cell growth and phenol contents after adding pyruvate (**B**), malate (**C**), succinate (**D**), and alanine (**E**). The chemicals contained the same number of carbon atoms (0.02 moles). (**A**) Control. (**F**) Their breakdown rates at 20 h after introducing six amino acids.

Thus, the effects of the four carbon sources on phenol degradation and cell growth were evaluated. When cultivated in these organic acids, the capacity for phenol degradation was significantly improved, consistent with the enhancement of oxidative phosphorylation and the TCA cycle response to phenol stress using GIMME analysis of the model *i*NX811. However, the addition of fructose did not promote phenol degradation. As shown in Figure 6, after adding sodium pyruvate, *A. lwoffii* NL1 reached its highest biomass at 14 h, and phenol was completely degraded after 15 h. After adding malate, the growth period of *A. lwoffii* NL1 (12 h) was shortened, and the highest biomass increased by 0.3 at OD_600_. After adding succinate, *A. lwoffii* NL1 reached its highest biomass at 16 h, and phenol was completely degraded. Considering that alanine can be converted into pyruvate by oxidation, the effect of alanine on phenol degradation was investigated. After adding alanine, *A. lwoffii* NL1 quickly entered the logarithmic growth phase at 5 h and reached its highest biomass at 14 h. Simultaneously, phenol was completely degraded after 14 h. Alanine promoted phenol degradation; however, its PHPP results were different from those of sodium acetate. In silico cell growth generally increased with the uptake rates of alanine and phenol but did not increase at uptake rates of alanine <4 mM and phenol >14 mM. The limitation of cell growth seemed to be eliminated by increasing the alanine supply. These substances (serine, glycine, aspartate, cysteine, proline, and glutamate) were investigated and showed a similar PHPP mode to alanine (Figure S2I–E). After adding these substances, the phenol degradation rate did not reach 100% at 20 h. Four exogenous substances supporting phenol degradation were identified using the model *i*NX811. Malate addition resulted in the most significant improvements in phenol degradation and biomass formation.

## 3. Materials and Methods

### 3.1. Reconstruction of the Genome-Scale Metabolic Model

#### 3.1.1. Construction of the Draft Model

A draft model of *A. lwoffii* NL1 was constructed using automated construction using ModelSEED [82] and manual reconstruction based on homologous comparison. The reference models were from *A. baumannii* [15] and *A. baylyi* [20] according to their close phylogenetic relationships. Gene–protein–reaction associations of the reference organisms were acquired by the local sequence similarity search (BLASTp). The criteria for the BLASTp were identity > 40% and match length > 70% [83]. The model contents from the two methods were manually curated by deleting duplicate annotations and unifying the reaction formula. Cytoplasmic and extracellular compartments were considered in the draft model of *A. lwoffii* NL1. The transport proteins of *A. lwoffii* NL1 were reannotated using transporters in the Transporter Classification Database (TCDB) [84]. The refined model was transformed into a mathematical model on the MATLAB 2019a platform using the xls2model.m program in the COBRA toolbox [85].

#### 3.1.2. Biomass Formation

The macromolecular composition of *A. lwoffii* NL1 was referenced to that of *A. baumannii* AYE [18]. Details of the biomass composition are provided in Table S6. DNA, mRNA, and amino acid compositions were calculated from the genome and protein sequences of *A. lwoffii* NL1. The contents of free fatty acids [25] and phospholipids [26] were extracted from the literature.

The biomassPrecursorCheck program verified the formation of biomacromolecules in the draft model. Metabolic gaps in the draft model led to an inability to synthesize a few biomacromolecules. These gaps were identified using the gapAnalysis.m program and filled by adding missing biochemical reactions to the draft model. The reaction pool for gap filling was from the KEGG pathway. The genes related to these reactions were annotated by comparing the proteomic data of *A. lwoffii* NL1 with the protein sequences in UniProtKB/Swiss-Prot [86].

### 3.2. Model Simulation and Analysis

#### 3.2.1. Cell Growth Simulation

Biomass formation was set as an objective function to simulate cell growth. Flux balance analysis (FBA) [87] was used to optimize the biomass equation using the GUROBI 8.0.0 (Gurobi Optimization Inc., Houston, TX, USA) mathematical optimization solver [88] on the MATLAB interface. Genes required for biomass biosynthesis were defined as essential genes and were analyzed on Luria–Bertani and minimal medium using succinate as the sole carbon source (see Table S7). The in silico essential genes (see Table S5) were evaluated by comparison with previously reported transposon insertion mutants of *Acinetobacter* [89,90] and the Database of Essential Genes (DEG) [91].

#### 3.2.2. Prediction of Drug Targets

Metabolites related to essential genes extracted by gene–protein–reaction associations in the model *i*NX811 were considered to be essential metabolites. The EMFilter framework reduces the anticipated important metabolites to a manageable quantity for in silico drug targets [18]. Common metabolites, such as cofactors and intermediates in the tricarboxylic acid (TCA) cycle, were excluded from the essential metabolites. Metabolites that coexist in humans were excluded to avoid drug side effects [92]. In addition, the drug targets should be endogenous metabolites of *A. lwoffii* that contain at least two consumption reactions.

#### 3.2.3. Context-Specific Metabolic Models

Transcriptomic datasets (SRR25444162, SRR25444164, and SRR25444166) under three conditions were integrated into the model *i*NX811 using the Gene Inactivity Moderated by Metabolism and Expression (GIMME) algorithm [78]. The three cultivation conditions were NaAc as the sole carbon source and NaAc with 0.5 or 1.5 g/L phenol as an additional carbon source (see Table S7). The first quartile of expression values of the metabolic genes was 13.04, 9.89, and 3.35 under the three conditions, respectively. The expression values of each gene above and below the expression threshold represented its presence (1) or absence (0) in the model *i*NX811 [93]. Three context-specific metabolic models were generated using CreateTissueSpecificModel.m (see Supplementary File S4). Feasible metabolic states of the models were explored by random sampling, and then the feasible flux distribution for each reaction with/without transcriptional constraints was plotted using plotSampleHist.m. Reaction fluxes (x values) of gimmeSolution were calculated using solveGimme.m. Reactions with a flux change (added phenol/without phenol) greater than twofold or less than one-half were thought to differ considerably with phenol concentration, and the reaction flux was visualized using the pheatmap package (https://cran.r-project.org/web/packages/pheatmap/index.html (accessed on 20 August 2023)) on the R platform (4.0.5).

#### 3.2.4. Phenotype Phase-Plane (PHPP) Analysis

PHPP analysis was used to calculate all possible variations in the two constraining variables to investigate the effects of substrate variables on cell growth [94]. The uptake rates of phenol (*x*-axis) and other substrates (*y*-axis) varied from 0 to 20 mmol/g dry cell weight/h. The in silico cell growth was plotted along the z-axis. All points on the *x*-*y* plane represent the optimal cell growth allowable for each pair of substrate uptake rates.

### 3.3. Model Validation

#### 3.3.1. Microbial Cultivation

The *A. lwoffii* NL1 used in this study was preserved in the China Center for Type Culture Collection (CCTCC NO: M2014329). *A. lwoffii* NL1 from a Luria broth (LB) agar slant was inoculated in 5 mL of liquid LB medium. Overnight cultures of *A. lwoffii* NL1 were collected and washed with sterile phosphate-buffered saline to achieve an optical density at 600 nm (OD_600_) of 0.5. Culture in liquid minimal mineral (MM) medium supplemented with specific carbon sources was performed at 28 °C and 200 rpm.

#### 3.3.2. Cell Growth and Phenol Utilization

Aliquots of bacterial suspension were inoculated (2% *v*/*v*) in test tubes containing MM supplemented with an individual carbon and nitrogen source. Cell growth was observed at 36 h to determine the utilization of each carbon and nitrogen source. The molar masses of the individual carbon and nitrogen sources were equal to those of phenol (0.5 g of phenol and 1.0 g of NH_4_Cl, respectively). The carbon sources included glucose, fructose, xylose, arabinose, mannitol, glycerol, ethanol, acetate, citrate, succinate, malate, phenol, 4-hydroxybenzoic acid, salicylate, benzoic acid, toluene, mandelate, benzene, phthalate, alanine, glycine, proline, serine, arginine, glutamate, glutamine, aspartate, threonine, valine, cysteine, tryptophan, methionine, leucine, phenylalanine, histidine, and lysine. The nitrogen sources included nitrate, NH_4_Cl, and urea.

In another series of experiments, aliquots of bacterial suspension were inoculated (2% *v*/*v*) in 20 mL of MM medium containing 0.5 g/L phenol and another substrate (0.02 moles of carbon) in 100 mL flasks. The effects of fructose, pyruvate, succinate, malate, and alanine on cell growth and phenol degradation were investigated by determining the OD_600_ of the biomass and the OD_510_ of the supernatants every few hours via a modified 4-aminoantipyrine colorimetric method [95]. Phenol concentration was also measured 20 h after adding serine, glycine, aspartate, cysteine, proline, or glutamate to 20 mL of MM containing 0.5 g/L phenol.

## 4. Conclusions

In this study, we constructed the first genome-scale metabolic model of *A. lwoffii* NL1 by combining manual and semi-automatic construction. The model *i*NX811 accurately predicted growth characteristics in different cultures. *A. lwoffii* NL1 showed multiple drug resistances and could effectively degrade phenol. Therefore, we used the model for antimicrobial systems and phenol biodegradation in *A. lwoffii*. Essential metabolite analysis predicted 12 potential target compounds for cell wall, lysine, vitamin, and fatty acid biosynthesis. Integrating transcriptomic data with model *i*NX811 provides a fundamental understanding of metabolic changes in response to phenol stress. These changes focused on central carbon metabolism, including enhancing oxidative phosphorylation, the TCA cycle, the oxaloacetate anaplerotic reaction, and the decreased glyoxylate cycle outflow from malate to pyruvate. A few transporters, such as salicylate exporters and oxygen uptake, were strengthened. In addition, the relationship between exogenous substances, phenol uptake, and cell growth was evaluated using PHPP analysis. Succinate, malate, alanine, and pyruvate enhanced phenol degradation in flask experiments.

## Figures and Tables

**Table 1:** Growth phenotypes of *A. lwoffii* NL1 on sole carbon and nitrogen sources.

Substrates		In Silico	In Vivo
Saccharides	Glucose	-	-
	Fructose	+	+
	Xylose	-	-
	Arabinose	-	-
Alcohol	Mannitol	-	-
	Glycerol	+	-
	Ethanol	+	+
Carboxylic acids	Acetate	+	+
	Citrate	+	+
	Succinate	+	+
	Malate	+	+
Aromatic xenobiotics	Phenol	+	+
	4-Hydroxybenzoic acid	+	+
	Salicylate	+	+
	Benzoic acid	+	+
	Toluene	+	+
	Mandelate	+	+
	Benzene	+	+
	Phthalate	+	+
Amino acids	L-Alanine	+	+
	Glycine	+	+
	Proline	+	+
	Serine	+	+
	Arginine	+	+
	Glutamate	+	+
	Glutamine	+	+
	Aspartate	+	+
	Threonine	+	+
	Valine	+	+
	Cysteine	+	+
	Tryptophan	-	-
	Methionine	-	-
	Leucine	+	+
	Phenylalanine	+	+
	Histidine	-	-
	Lysine	-	-
Nitrogen sources	NaNO_3_	-	-
	NH_4_Cl	+	+
	Urea	+	+

The symbol + represents cells that can thrive only on this kind of carbon source or nitrogen source. The symbol - refers to cells that cannot grow only on this kind of carbon source or nitrogen source.

**Table 2:** Drug target prediction according to essential metabolite analysis.

Pathway	Essential Metabolites	Enzymes	Genes
Amino sugar and nucleotide sugar metabolism	alpha-D-Glucosamine 1-phosphate	D-Glucosamine 1,6-phosphomutase, glucosamine-1-phosphate-acetyltransferase	*LNSL_0126 LNSL_2808*
Lipopolysaccharide biosynthesis	D-arabinose-5-phosphate	D-Arabinose-5-phosphate isomerase, 3-deoxy-8-phosphooctulonate synthase	*LNSL_ 1272* *LNSL_ 1680*
Polysaccharide biosynthesis	dTDP-glucose	dTDP-Glucose 4,6-hydro-lyase, dTDP-glucose 4-epimerase, dTDP-glucose synthase	*LNSL_2851* *LNSL_2820* *LNSL_2850*
Starch and sucrose metabolism	Trehalose 6-phosphate	Trehalose 6-phosphate synthase	*LNSL_0763*
Lysine biosynthesis	Dehydrodipicolinate	Dehydrodipicolinate synthase, dihydrodipicolinate reductase	*LNSL_ 0056* *LNSL_ 2865*
	N-Succinyl-LL-2,6-diaminoheptanedioate	Succinyldiaminopimelate transaminase, succinyl-diaminopimelate desuccinylase	*LNSL_ 1115* *LNSL_ 2239*
	meso-2,6-Diaminoheptanedioate	Diaminopimelate epimerase, UDP-N-acetylmuramoylalanyl-D-glutamate-2,6-diaminopimelate ligase	*LNSL_ 2102* *LNSL_ 2621*
	L-Aspartate 4-semialdehyde	Aspartate-semialdehyde dehydrogenase, 4-hydroxy-tetrahydrodipicolinate synthase	*LNSL_0359* *LNSL_ 0056*
Folate biosynthesis	4-Aminobenzoate	4-Amino-4-deoxychorismate pyruvate-lyase, dihydropteroate synthase	*LNSL_2017* *LNSL_2140*
	2-Amino-4-hydroxy-6-hydroxymethyl-7,8-dihydropteridine	7,8-Dihydroneopterin aldolase, dihydropteroate synthase	*LNSL_1717* *LNSL_2140*
	Chorismate	Aminodeoxychorismate synthase	*LNSL_0563*
Thiamine metabolism	1-Deoxy-D-xylulose 5-phosphate	1-Deoxy-D-xylulose-5-phosphate synthase	*LNSL_2536*
Vitamin B6 metabolism	1-Deoxy-D-xylulose 5-phosphate	Pyridoxine 5′-phosphate synthase	*LNSL_2003*

## Data Availability

Data and materials are available from the corresponding author.

## Supplementary Materials

The supporting information can be downloaded at https://www.mdpi.com/article/10.3390/ijms25179321/s1.
