# Supplementary material for: Reconstruction and Analysis of a Genome-Scale Metabolic Model of Acinetobacter lwoffii
Source: Int J Mol Sci. 2024 Aug 28;25(17):9321. doi: 10.3390/ijms25179321 (PMC11395192; doi:10.3390/ijms25179321)
Supplement: Supplementary file 1 [file ijms-25-09321-s001.zip › Supplementary File S5.pdf]

Supplementary File S5

Reconstruction and analysis of a genome-scale metabolic model of *Acinetobacter lwoffii*

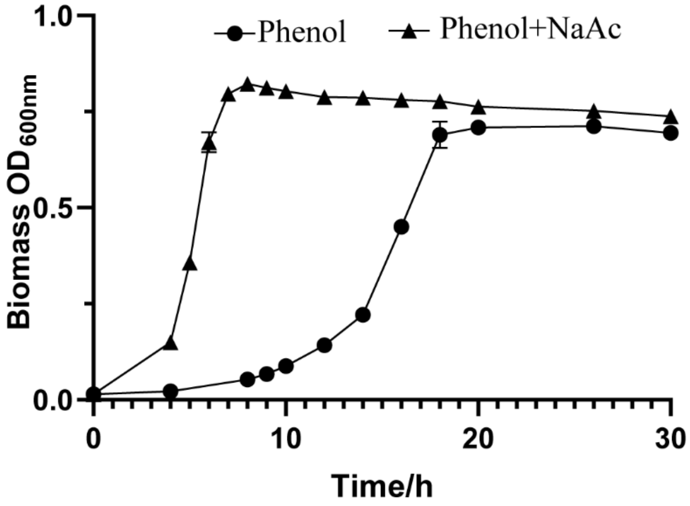

Figure S1. Cell growth of *A. lwoffii* on different medium
The circle denotes phenol medium. The triangle denotes phenol medium adding sodium acetate.

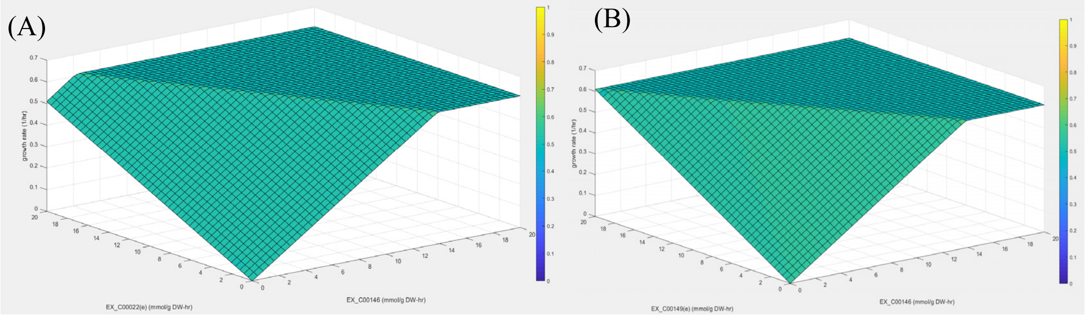

(C)

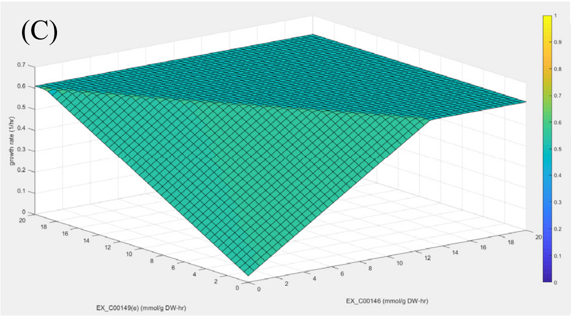

(D)

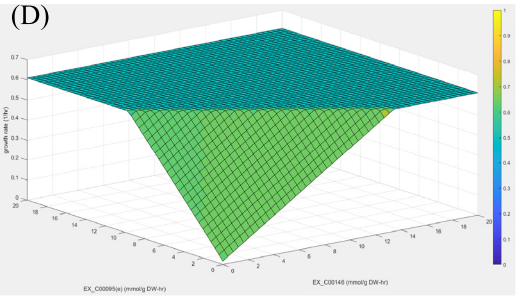

(E)

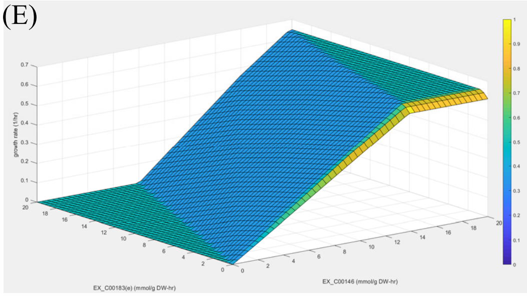

(F)

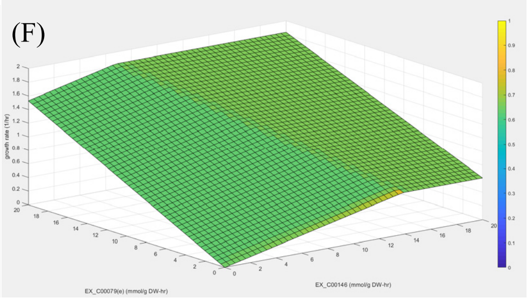

(G)

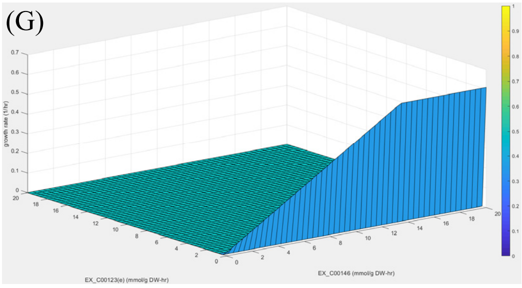

(H)

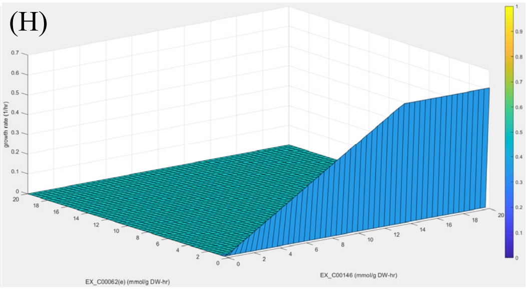

(I)

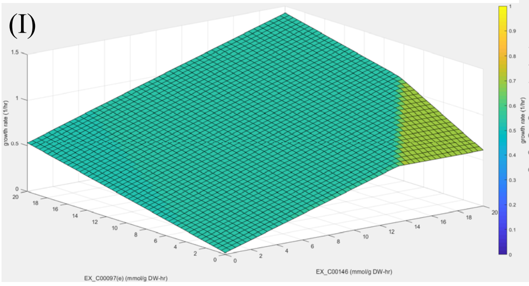

(J)

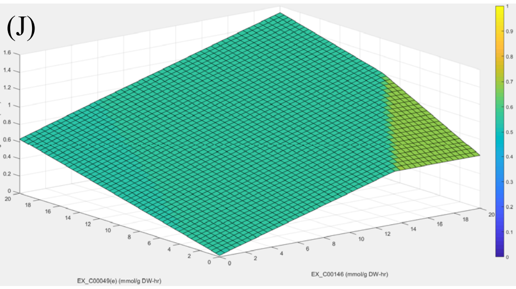

(K)

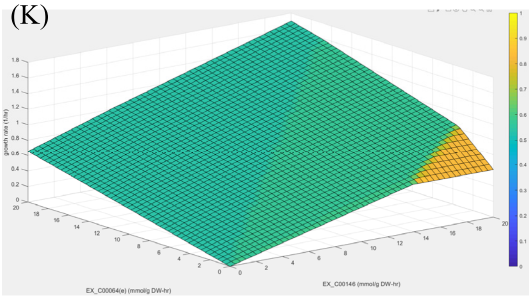

(L)

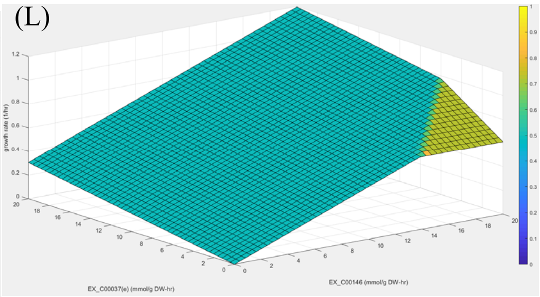

(M)

(N)

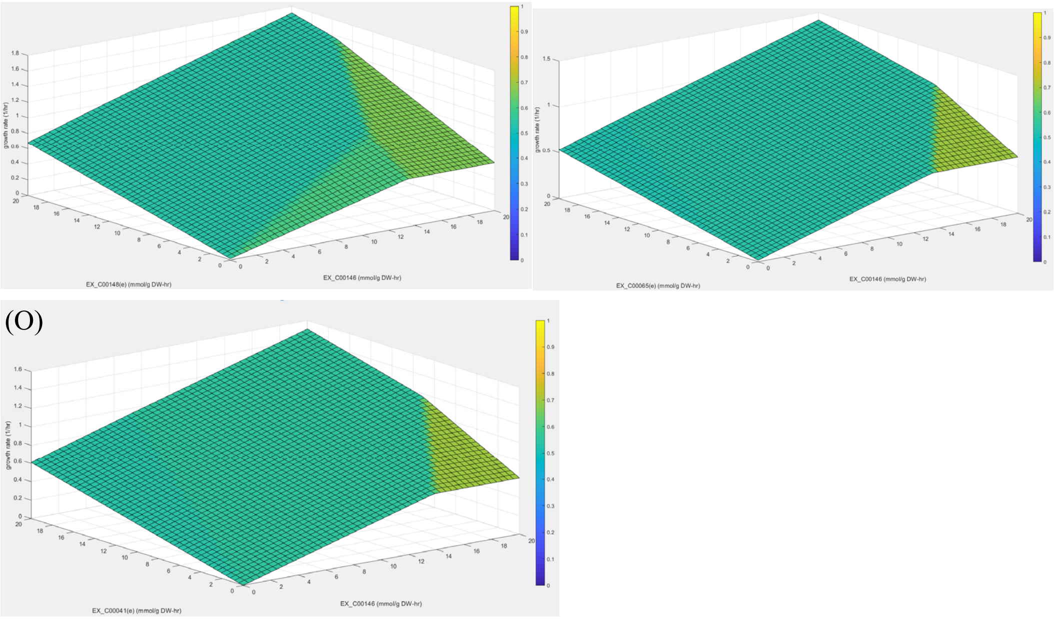

Figure S2. The effects of phenol and other different substrate on cell growth using phenotype phase plane analysis. The label (A-O) respectively represent L-Malate, pyruvate, succinate, D-fructose, L-Valine, L-Phenylalanine, L-Leucine, L-Arginine, L-Cysteine, L-Aspartate, L-Glutamate, Glycine, L-Proline, L-Serine, and L-Alanine.
